# Whole-Genome Sequence of *Francisella noatunensis* subsp. *orientalis* Strain FNO01 Isolated from Diseased Nile Tilapia in Brazil

**DOI:** 10.1128/genomeA.01603-15

**Published:** 2016-01-21

**Authors:** H. C. P. Figueiredo, C. A. G. Leal, F. L. Pereira, S. C. Soares, L. A. Gonçalves, F. A. Dorella, A. F. Carvalho, V. A. C. Azevedo

**Affiliations:** a AQUACEN, National Reference Laboratory for Aquatic Animal Diseases, Ministry of Fisheries and Aquaculture, Federal University of Minas Gerais, Minas Gerais, Brazil; b Laboratory of Cellular and Molecular Genetics, Institute for Biological Science, Federal University of Minas Gerais, Minas Gerais, Brazil

## Abstract

This paper describes the complete genome sequence of *Francisella noatunensis* subsp. *orientalis* strain FNO01, which was isolated during the first outbreak of francisellosis in cultured Nile tilapia in Brazil. The genome is composed of a circular chromosome with 1,859,830 bp and a G+C content of ~32%.

## GENOME ANNOUNCEMENT

*Francisella noatunensis* subsp. *orientalis* (1) is recognized as one of the most important pathogens of cultured Nile tilapia (*Oreochromis niloticus*) (2). *F. noatunensis* subsp. *orientalis* is the etiologic agent of pyogranulomatous and granulomatous infections in fish. In the last few years, *F. noatunensis* subsp. *orientalis* has been responsible for a large number of deaths of tilapia and other freshwater fish species cultured in the United States, United Kingdom, Japan, Taiwan, Jamaica, Costa Rica, and some other Latin American countries (3, 4). This species is a Gram-negative pleomorphic bacterium and is strictly aerobic and nonmotile. This sequencing initiative is a part of a large study on the molecular epidemiology of *F. noatunensis* subsp. *orientalis* in Brazil.

DNA from *F. noatunensis* subsp. *orientalis* strain FNO01 was isolated from overnight culture with the Maxwell 16 tissue DNA purification kit using the Maxwell 16 system (both from Promega, USA). The genome was sequenced with the Ion Torrent PGM sequencing system (Life Technologies, USA) using a 200-bp-fragment library kit, according to the manufacturer’s recommendations. The obtained depth coverage of ~381-fold was assembled using Newbler 2.9 (Roche, USA), with the recommended parameters. These processes resulted in assembly with 14 contigs and an *N*_50_ of 303,981 bp. These contigs were scaffolded through the CONTIGuator 2.0 software (5), using *F. noatunensis* subsp. *orientalis* strain FNO12 (accession no. CP011921) as a reference. In-house scripts were used to evaluate overlapped contigs, and remaining gaps were closed with CLC Genomics Workbench version 7.0 (CLC bio, Germantown, MD) by filling with successive mappings in the gap flanks, until the overlaps were found.

Strain FNO01 is composed of a circular chromosome with a length of 1,859,830 bp and a G+C content of 32.30%. The genome was annotated using an in-house transfer script, using the FNO12 strain as a reference.

### Nucleotide sequence accession number.

This whole-genome project has been deposited at GenBank under the accession number CP012153.
